# Clinical Pharmacist-Led Interventions for Improving Breast Cancer Management—A Scoping Review

**DOI:** 10.3390/curroncol31080312

**Published:** 2024-07-25

**Authors:** Radiana Staynova, Evelina Gavazova, Daniela Kafalova

**Affiliations:** Department of Organisation and Economics of Pharmacy, Faculty of Pharmacy, Medical University of Plovdiv, 4002 Plovdiv, Bulgaria; evelina.gavazova@mu-plovdiv.bg (E.G.); daniela.kafalova@mu-plovdiv.bg (D.K.)

**Keywords:** breast cancer, pharmaceutical intervention, pharmacist, oncology, quality of life, patient education, adherence

## Abstract

Breast cancer is the leading cause of cancer-related death in women worldwide and the fifth most common cause of cancer death overall. Most women with breast cancer have a good prognosis if the cancer is detected at an early stage and the patients have access to the appropriate treatment and disease management. This study aims to evaluate the impact of pharmacist-led interventions on breast cancer management and health outcomes. A literature review was carried out through the scientific databases PubMed, Scopus, and Web of Science using predefined keywords. Only full-text original articles written in English that investigated the role of the pharmacist in the management of breast cancer were included in the final analysis. No publication date limits were set. A total of 1625 articles were retrieved from the electronic databases, of which 14 met the inclusion criteria. The current scoping review consists of different study types, including randomized controlled trials, cross-sectional studies, pre-post studies, retrospective cohort studies, quality improvement projects, case-control studies, and one pharmacoeconomic study. Pharmacists commonly provided the following interventions: consultations regarding chemotherapy treatment, risk assessment and patient education, adverse drug reactions and drug-drug interactions detection, and adherence assessment. This scoping review highlights the beneficial effects of the involvement of pharmacists in breast cancer management, such as better quality of life, reduced drug interaction risk, greater adherence rates, and improved patient knowledge. This confirms the importance of including the pharmacist in the oncology team caring for patients with breast cancer.

## 1. Introduction

Women’s health is a critical aspect of overall well-being, yet there is often a tendency to neglect it due to societal expectations and roles [1]. Breast cancer poses a significant challenge and is the leading cause of cancer-related death among women [2]. According to data from the World Agency for Research on Cancer (IARC) in 2020, about 2.26 million women were diagnosed with breast cancer, and almost 685,000 deaths were caused by the disease globally [2]. Despite the multiple effective therapeutic options, breast cancer is the leading cause of cancer-related death in women worldwide and the fifth most common cause of cancer death overall [3]. Late diagnosis is a prevailing issue, resulting in reduced treatment efficacy and higher mortality rates [4]. Most women with breast cancer have a good prognosis if the cancer is detected at an early stage and the patients have access to the appropriate treatment. Chemotherapy remains the standard of care for this type of disease, although complementary and alternative treatment methods are also described in the literature [5,6]. Despite remarkable progress in this setting to date, some patients will see their disease return in the long term, which is why there is a critical need to continue to optimize early breast cancer care and to take additional measures to prevent the disease from evolving into an advanced, incurable stage [7].

Breast cancer treatment is a complex and multi-step process, the outcome of which depends on the cooperation and coordination between different healthcare providers. After diagnosis, patients need to be provided with detailed chemotherapy education, including the mechanism of action of chemotherapy agents, treatment goals, possible side effects, and symptom management [8]. Therefore, according to the current recommendations of the European Association of Medical Oncology (ESMO), the treatment should be carried out by a multidisciplinary team consisting of a medical oncologist, a surgeon, a radiation therapist, a pathologist, and a specialized (best in breast cancer) oncology nurse [9]. Patients should be actively involved in all management decisions during the therapeutic process. The result of the healthcare team’s efforts depends to a large extent on the involvement of the patients and their clear understanding and support of the jointly selected therapeutic decisions [10].

For more than 50 years, oncology pharmacists have proved to be core multidisciplinary team members in the care of patients with cancer [11]. Recent studies confirm oncology pharmacists’ contribution to better patient outcomes, including a reduced number of drug-related problems (DRPs), improved quality of life (QoL), and increased adherence rates [12,13,14]. Additionally, pharmacist-led interventions in oncology care have been reported to be cost-effective and cost-saving [15,16]. The role and activities of oncology pharmacists are continuously expanding, and now this role is focused on patient-centered care [8,11]. Common roles for oncology pharmacists, their clinical functions, and different settings for professional development, have been comprehensively discussed by Holle et al. [11]. One of the key activities provided by oncology pharmacists is patient counseling and education [17].

Oncology pharmacists meet and monitor the patient throughout the course of treatment to assess and reduce the potential for adverse drug reactions (ADRs). This helps build a relationship between pharmacists and cancer patients and can be a key element in identifying ADRs as well as ensuring medication adherence [18]. The results of a recent interventional study show a significant improvement in medication adherence rates after education and counseling provided by pharmacists [19].

**Figure 1:**
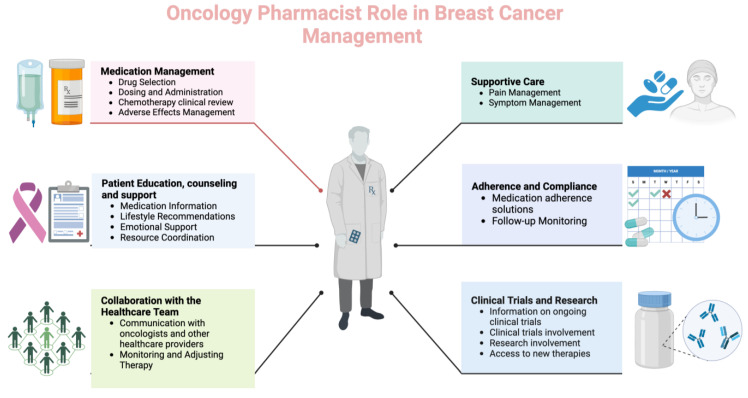
Different roles and functions of oncology pharmacists in breast cancer care (Created with BioRender.com, accessed on 23 April 2024).

Pharmacists can play a crucial role in managing patients with breast cancer as part of the healthcare team. Their responsibilities encompass various aspects of pharmaceutical care, including medication management, education, and support (Figure 1).

Over the years, in many countries, clinical pharmacists have become an integral part of the oncology team involved in the treatment of breast cancer. Oncology pharmacists have expert knowledge of medications used in the treatment of breast cancer, including their pharmacokinetic properties. As essential multidisciplinary team members, oncology pharmacists help optimize the benefits of drug therapy and help reduce adverse and toxic effects [18]. Integrating oncology pharmacists into healthcare teams adds value through their expertise in medication management and enhancing adherence, allowing physicians to optimize time during clinic appointments and potentially see more patients [20].

To the best of our knowledge, no systematic review has assessed the clinical pharmacist’s role in caring for breast cancer patients. Therefore, this study aims to evaluate the impact of pharmacist-led interventions on breast cancer management and health outcomes.

## 2. Materials and Methods

### 2.1. Search Strategy and Selection Criteria

**Figure 2:**
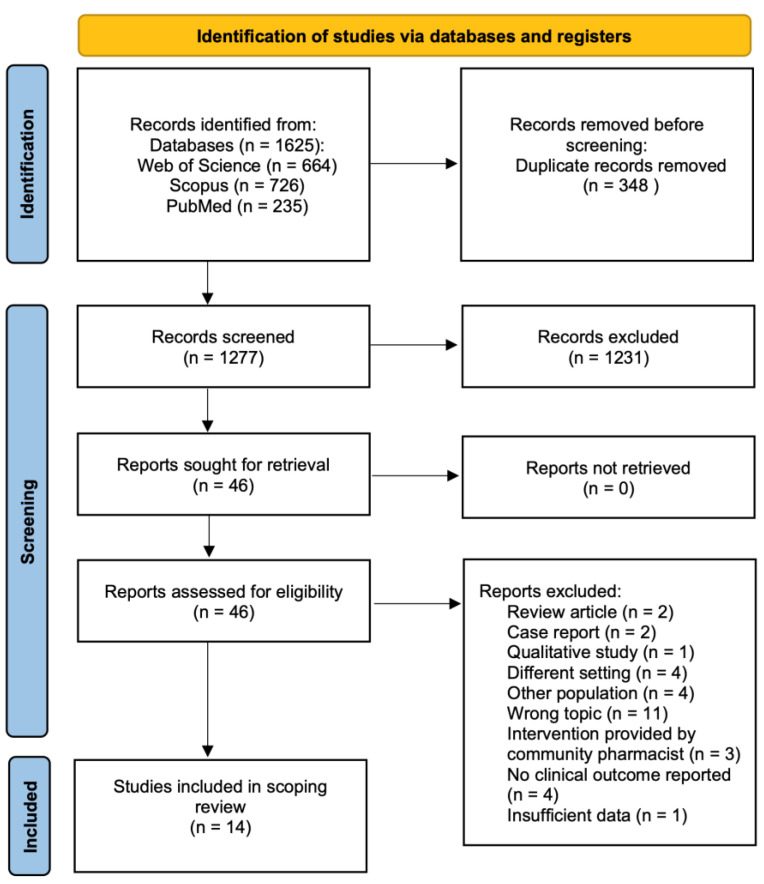
PRISMA flowchart for study selection.

A systematic search was conducted on the PubMed, Web of Science, and Scopus databases. The following keywords were used: (“pharmacist” OR “clinical pharmacist” OR “oncology pharmacist”) AND (“breast cancer”). The initial search for relevant articles started in December 2023 and was finalized in March 2024. No limitations on the date of published articles were set. The current scoping review was carried out in accordance with the Preferred Reporting for Systematic Reviews and Meta-Analyses (PRISMA) guidelines (Figure 2) [21].

### 2.2. Eligibility Criteria

Inclusion criteria were full-text research articles written in English that investigate the impact of pharmacist-led interventions on health outcomes in breast cancer patients (e.g., quality of life, drug interaction risk, adherence rate, patient knowledge, etc.). Non-English-language articles and those that did not report on the primary outcome of the current study were excluded from the analysis. Systematic reviews, meta-analyses, narrative reviews, case reports, qualitative studies, editorials, letters to the editor, commentaries, and abstracts from conferences were not considered for inclusion in this review. The eligibility criteria of articles based on the PICO framework (population, intervention, comparison, and outcome) are presented in Table 1.

### 2.3. Data Extraction

One author (R.S.) performed the initial search in the selected databases. In the next stage, duplicates were removed using Zotero software v. 6.0.37. Two authors (R.S. and E.G.) independently screened the titles and abstracts of identified articles to exclude ineligible studies. The full text of papers that met the inclusion criteria was retrieved and assessed by one author (R.S.) and rechecked by a second author (E.G.). In case of any discrepancies, they were addressed following a discussion with a third author (D.K.).

The following relevant data from each publication that met the inclusion criteria were extracted:

(1) Primary author and year of publication
(2) Country
(3) Study design
(4) Sample size
(5) Objective
(6) Study outcomes and main results

## 3. Results

A total of 1625 articles were retrieved from the electronic databases in the initial search, of which 14 met the inclusion criteria and assessed pharmacist interventions on health outcomes in breast cancer patients (Table 2) [13,22,23,24,25,26,27,28,29,30,31,32,33,34]. After the removal of duplicates, the remaining 1277 papers were screened by title and abstract. Following the screening process, 1231 records were excluded due to their irrelevance to the main topic of this review. The full texts of the remaining articles were assessed for eligibility. Of those, 32 were removed for several reasons, as illustrated in Figure 2.

### 3.1. Characteristics of Studies Included

Of the 14 studies included in the review, the majority were interventional, including pre-post studies (n = 5, 35.7%) [13,22,23,24,25], randomized controlled studies (n = 1, 7.1%) [26] and prospective clinical trials (n = 1, 7.1%) [27], followed by observational studies, including case-control studies (n = 2, 14.3%) [30,34], cross-sectional studies (n = 2, 14.3%) [28,33] and retrospective cohort studies (n = 1, 7.1%) [32], and the remaining were quality improvement projects (n = 1, 7.1%) [31] and economic evaluations (n = 1, 7.1%) [29]. Regarding country of origin, five of the included studies were conducted in the USA [13,23,26,31,32], three in Japan [25,28,29], one in Egypt [24], India [22], France [27], Brazil [33], Malaysia [30], and Iraq [34]. The studies were published between 2012 and 2023. The pharmacist’s intervention involved mainly one or more of the following: pharmacist-led counselling or education [24,25,28,29,30] and management of medication adherence, ADRs and/or drug-interactions assessment [13,23,25,26,27,31,32,33,34]. The sample size of breast cancer patients in each study ranged from eighteen [23] to one-hundred forty-five [32]. Three of the studies reported health outcomes in patients diagnosed with different types of malignancies, including breast cancer [13,26,33]. For the purpose of our study, only the results related to breast cancer patients were extracted and analyzed.

### 3.2. Patient Education and Counselling

Six studies reported pharmacist engagement in patient education and counselling [22,24,25,28,29,30]. Of those, four studies explored the effect of pharmacist counselling on quality of life (QoL) in patients with breast cancer [24,28,29,30]. These studies are analyzed in the ‘Improving Quality of Life (QoL)’ section presented below.

The results from a pre-post study conducted in Japan revealed that pharmacists spent 75 h per month in patient education and ADRs monitoring, which led to the reduction of ADRs (nausea and vomiting) and facilitated the rational use of anti-emetic drugs [25].

The beneficial effect of pharmacist-led intervention on patient knowledge was also demonstrated by Dang et al. [30]. Pharmacist-led pre-chemotherapy counselling led to improvements in patient knowledge scores and a better understanding of the chemotherapy regimen and side effects [30].

### 3.3. Adherence Assessment

Four of the included studies assessed the change in adherence rate after pharmacist-led interventions [13,23,31,34]. A study conducted in the USA by Muluneh et al. described an innovative model involving the integration of a closed-loop, pharmacy-led oral chemotherapy management program at ambulatory oncology clinics [13]. In this closed-loop model, clinical pharmacists specializing in oncology were integrated into breast oncology clinics to manage patients receiving oral chemotherapy. One of the activities of clinical pharmacists was to assess and enhance the adherence to oral chemotherapy, which was performed at every patient encounter. For measuring adherence, patient self-reports and medication possession ratio (MPR) were used. After pharmacist-led interventions, patients achieved a self-reported adherence rate of 86%, which was verified by the MPR calculation of 85% (the goal adherence rate was >80%) [13].

Another study conducted in the USA aimed to assess the feasibility of an intervention for symptom monitoring and management, which clinical pharmacists facilitated to improve adherence to breast cancer adjuvant endocrine therapy (AET) [23]. In this pilot study, clinical pharmacists used guideline-based symptom management and adherence-supporting tools with nonadherent breast cancer patients. In the six-month study period, 44% of patients became adherent. Despite its small sample size (only 18 patients), this intervention has promising potential to enhance support and self-efficacy and improve patient symptoms and adherence rates [23].

A recent quality improvement project describes different interventions provided by outpatient clinical pharmacists for improving management and adherence to oral cancer therapy [31]. A total of 31 adherence counseling interventions were documented, including different methods such as medication calendars and increased monitoring frequency. Other pharmacist-led interventions included in this project consist of medication reconciliations and clinical recommendations (e.g., therapy modification, dose adjustments, toxicity management, and monitoring). The findings from this project indicate that the incorporation of a clinical pharmacist in a multidisciplinary team was associated with decreased treatment day delays (from 7.7 days in the preintervention assessment to 2.1 days after the implementation of the program, *p* < 0.001) [31].

An Iraqi study measured the adherence rate after pharmacist-led intervention in women with breast cancer on adjuvant oral hormonal therapy [34]. The researchers used the Morisky Medication Adherence Scale eight (8) items (MMAS-8) and the Beliefs about Medication Questionnaire (BMQ) for measuring adherence to medication therapy and assessing women’s beliefs. Two months after the initiation of the intervention, 65.4% of the patients in the pharmacist-led group demonstrated apparent adherence to adjuvant oral hormonal therapy. In addition, the pharmacist intervention significantly improved the necessity beliefs and necessity-concern differential when compared with the control group [34]. 

### 3.4. Management of Adverse Side Effects and Drug Interactions

Five studies reported the impact of pharmacist involvement on the detection and management of ADRs, drug-drug interactions (DDIs), and other DRPs [25,26,27,32,33]. One study reported that clinical pharmacist interventions facilitated a decrease in chemotherapy-induced nausea and vomiting. Additionally, pharmacist activities were cost-effective and reduced anti-emesis costs by 16% [25]. In a randomized clinical trial, older cancer patients received a multidisciplinary geriatric assessment-driven intervention that generated a significant reduction in chemotherapy-related toxic effects. In this RCT, pharmacists were involved in the detection of inappropriate polypharmacy and its rectification (e.g., providing recommendations for DDIs, potentially inappropriate medications, therapeutic duplication, etc.) [26].

In a recent prospective study, researchers from France presented the results of a comprehensive medication history carried out by a hospital pharmacist who participated in a clinical trial to determine the risk of DDIs. Pharmacist-led interventions have reduced the risk of DDIs in almost one-third of patients [27].

A pharmacist-led anticoagulation management service in breast cancer clinics has shown that, compared to other studies conducted on cancer patients receiving regular medical care, the pharmacy service reduced the frequency of recurrent venous thromboembolism and increased the time spent on therapeutic International Normalized Ratios (INRs) [32].

Ferracini et al. reported that pharmacist-led interventions had a significant impact on avoiding prescribing errors. In this cross-sectional study, clinical pharmacists monitored drug therapy and analyzed 1874 prescriptions from 248 hospitalized patients with gynecological and breast cancer. Of the 1874 evaluated prescriptions, 215 (11.5%) contained at least one error. Pharmacists provided 294 pharmaceutical interventions, including drug interaction assessment and dose adjustment, of which 73.5% were accepted by oncologists [33].

### 3.5. Improving Quality of Life (QoL)

Four of the analyzed articles evaluated the effect of pharmacist consultations and interventions on the QoL in patients with breast cancer [22,24,28,29].

Tanaka et al. applied the Quality-of-Life Questionnaire for Cancer Patients Treated with Anticancer Drugs (QOL-ACD) to evaluate the impact of pharmacist counseling on breast cancer patients who underwent their initial course of outpatient cancer chemotherapy [28]. The QOL-ACD instrument is a generic questionnaire that was developed in Japan by Kurihara et al. for measuring QoL in patients diagnosed with different types of cancer who are treated with antineoplastic drugs [35]. Findings from this study revealed that pharmacists can improve patients’ QoL regarding malaise and nausea by providing personal counseling before medical examinations [28].

The same authors performed a pharmacoeconomic analysis to assess the utility of pharmacist counseling care for breast cancer chemotherapy outpatients compared with standard care [29]. The researchers used the EQ-5D instrument to evaluate the QoL. Results from this study revealed that pharmacists’ counseling in patients with breast cancer led to improved patient QoL with an acceptable incremental cost-effectiveness ratio. As a limitation of this study, it can be mentioned that its small sample size consisted of 38 patients, of whom 19 received pharmacist consultation [29].

Another study also applied the EQ-5D-5L instrument to determine quality-adjusted life-years (QALYs) for measuring humanistic outcomes after the provision of oncology pharmacist services to patients with breast cancer [22]. Services delivered by oncology pharmacists in this study included drug-related information provided to oncologists, medication chart reviews for verifying the appropriateness of anti-neoplastic medications, counseling on the usage of medications, etc. Pharmacists’ involvement after the administration of three chemotherapy cycles led to significantly improved QALY in the intervention group compared to the control group [22].

The fourth study was conducted in Egypt, and the effect of the pharmacists’ interventions on QoL was assessed using the EORTC QLQ-BR23 questionnaire [24]. The results obtained by Farrag et al. demonstrated a significant overall improvement in the QOL regarding functional and symptom scales (*p* < 0.001) of the EORTC QLQ-BR23 instrument after 6 months of follow-up and the involvement of a clinical pharmacist [24].

## 4. Discussion

This scoping review highlighted four key themes related to clinical pharmacist-led interventions for improving breast cancer management: patient education and counseling, adherence assessment, management of adverse side effects and drug interactions, and improving QoL.

Patient education is an essential element in the treatment of cancer patients and is vital to the success of oral treatment [36]. The transition from intravenous delivery of medications to oral therapy allows for more opportunities to improve the way of prescribing, dispensing, and monitoring the therapeutic process [18]. Oncology pharmacists are uniquely positioned to improve patient care in each of these areas [37]. They can play an important role in the education process of breast cancer patients by providing information on prescribed medications, potential side effects, drug interactions, and the importance of adherence [38]. Moreover, pharmacists may offer recommendations on lifestyle modifications, such as dietary considerations, exercise, and smoking cessation [39]. In addition to their undeniable role in patient education, interventions provided by oncology pharmacists have been reported to be associated with improved patient knowledge and higher satisfaction levels [13,14,40,41]. A systematic review conducted by Segal et al. confirms the impact of the oncology pharmacist on patient satisfaction [14]. Furthermore, this is demonstrated in a study by Mulunesh et al. in which patient satisfaction with pharmacist education is rated “excellent” [13].

Adherence is another key component in the management of cancer patients undergoing oral chemotherapy. According to the WHO, poor adherence to long-term treatment is a major global problem, resulting in poor health outcomes and higher health costs [42]. Suboptimal medication adherence may lead to the recurrence of cancer, elevated rates of hospital admissions, and an increased risk of mortality [43]. Factors that hinder adherence include complex treatment regimes, side effects, higher medication costs, low health literacy, and limited patient knowledge [37]. Since 80% of patients with breast cancer have hormone receptor-positive tumors, AET is a common approach [44]. Nonadherence is a significant problem of AET in women with breast cancer [45,46,47]. Although AET offers positive health benefits, this therapy is associated with significant side effects that lead to poor medication adherence [42,44]. Studies have shown that treating and managing breast cancer in older women can be challenging. This is largely due to polypharmacy, which in turn causes DRPs and leads to poor adherence [48]. A recent systematic review has shown that between 30 and 60% of women take less of the prescribed AET than recommended, while between 30 and 70% of women prematurely stop their treatment at the end of the fifth year [49]. As medication experts, pharmacists can help fill the gaps in medication adherence in breast cancer patients by providing education and counselling, social support, and developing ongoing adherence assessments [50]. The pharmaceutical interventions described in the studies included in our scoping review demonstrated a measurable impact on adherence levels following pharmacist involvement. Moreover, clinical pharmacists used various aids to enhance medication adherence, such as calendars and schedules [31]. One of the included studies applied two validated questionnaires, MMAS-8 and BMQ, to measure medication adherence [34]. Several other studies evaluating the role of pharmacists in improving adherence among cancer patients have also reported using these tools [19,51,52]. Despite the limited number of studies evaluating the pharmacist’s role in improving adherence in breast cancer patients, this is an ongoing and promising area of research.

Oncology pharmacists are key specialists in the provision of up-to-date information to oncologists and other healthcare providers regarding antineoplastic medications, potential drug interactions, and adverse side effects [36]. Oncology pharmacists meet with patients shortly after a cancer diagnosis to gain a better understanding of their habits and current medications. Based on this information, pharmacists determine the risk of drug interactions and develop a patient-specific treatment plan [18]. Many published studies confirm the beneficial role of clinical pharmacists in the identification of DRPs in cancer patients [12,53,54,55]. Different tools, like DDI checkers, websites, and mobile applications, are reported to be used [56]. DRPs observed in studies involving patients with cancer include ADRs, DDIs, inappropriate medications, overdosing, untreated indications, contraindications, administered omissions, etc. [33,53,54,57]. In addition to the detection of DRPs, interventions delivered by pharmacists resulted in a significant reduction of ADRs [58]. The most commonly performed pharmaceutical interventions reported in the studies identified by our review include medication review, management of ADRs, patient education, dosage adjustment, treatment discontinuation, and therapeutic drug monitoring [25,26,27,32,33].

In addition to breast cancer survival, QoL is another important determinant in the overall process of disease management [59]. In the scientific literature, there are many research articles that assess the role of the pharmacist in improving the QoL of patients suffering from different chronic diseases, including cancer [60]. There are also a variety of studies measuring QoL in breast cancer patients [59,61,62]. However, only these four articles included in our scoping review evaluate both aspects. Various validated tools are available for measuring QoL, including those specifically developed for cancer patients. One of the studies included in our review assessed the effect of the pharmacist’s interventions on QoL using the EORTC QLQ-BR23 questionnaire [24]. The latter is a specific breast cancer instrument designed by the European Organization for Research and Treatment of Cancer (EORTC) [63]. This questionnaire was widely used in many studies for measuring QoL in patients with breast cancer [64,65,66,67]. Despite the encouraging findings demonstrated in the scoping review, future studies involving a larger sample size would confirm the benefits of pharmacist-led interventions on QoL in breast cancer patients. The availability of generic (e.g., EQ-5D or EQ-5D-5L) and specific (EORTC QLQ-BR23) instruments to assess the QoL of breast cancer patients would facilitate this process.

### Strengths and Limitations

We emphasize that this is the first scoping review that comprehensively synthesizes the current data on the role of clinical pharmacists in breast cancer management. Moreover, the search period was broad enough, without limitations on the date of published papers, and three major databases were investigated to find every relevant record.

It is important to highlight a few of this study’s limitations. Firstly, some of the studies reviewed had small sample sizes, which increased the risk of error (sampling bias). Secondly, only articles published in English were selected for inclusion. As a result, we may have missed important findings from studies published in other languages. Thirdly, the review only considered research reports with defined, measurable outcomes. Furthermore, a meta-analysis was not conducted due to the inclusion of many different study designs and the heterogeneity of the data.

## 5. Conclusions

This scoping review highlights the beneficial effects of the involvement of pharmacists in breast cancer management, such as better QoL, reduced drug interaction risk, greater adherence rates, and improved patient knowledge. Additionally, some pharmacist-led interventions were reported to be cost-effective or associated with a high level of patient satisfaction. This underscores the importance of incorporating a clinical pharmacist into the oncology team caring for patients with breast cancer. The findings summarized in the review could serve as a strong foundation for future research. More randomized controlled studies involving a larger sample of breast cancer patients are needed to confirm the clinical and economic benefits of pharmacist-led interventions.

## Figures and Tables

**Table 1:** PICOS criteria for study selection.

Parameter	Description
**P**opulation	Breast cancer patients (aged 18 years or older)
**I**ntervention	Any intervention with clinical/hospital pharmacist involvement—whether individually or as a member of the healthcare team
**C**omparison	Studies with or without a control group (e.g., standard care or no intervention)
**O**utcome	Improved quality of life, reduced drug interaction risk, increased adherence rate, and improved patient knowledge
**S**tudy design	Randomized controlled trials, cross-sectional studies, pre-post studies, retrospective cohort studies, quality improvement projects, case-control studies, interventional prospective studies, pharmacoeconomic studies

**Table 2:** Characteristics of included studies.

Author, Year	Country	Study Design	Sample Size	Objective	Study Outcomes and Main Results
Dang et al., 2016 [30]	Malaysia	Prospective case-control study	*n* = 38 breast cancer patients	Evaluation of the effectiveness of pharmacist-led pre-chemotherapy counseling on the knowledge of chemotherapy-treated breast cancer patients.	Pharmacist-led pre-chemotherapy counseling led to significant improvement in patient knowledge regarding chemotherapy regimen and its side effects.
Farrag et al., 2020 [24]	Egypt	Single-center interventional pre-post study	*n* = 60 breast cancer patients	Evaluation of pharmacist’s educational intervention on health outcomes and QoL in patients with breast cancer.	Clinical pharmacist interventions resulted in beneficial clinical outcomes in patients with breast cancer (reduction of treatment-related side effects and improvement of patients’ QoL).
Ferracini et al., 2018 [33]	Brazil	Cross-sectional, prospective study	*n* = 248 (106 patients with breast cancer)	Evaluation of types of prescribing errors, pharmaceutical interventions, and differences in clinical significance in prescriptions for hospitalized patients with breast and gynecological cancer.	A total of 294 pharmaceutical interventions were provided by clinical pharmacists. The most commonly used pharmaceutical interventions concerned drug interaction (30.3%) and dose adjustments (26.5%). Of all interventions, 73.5% were accepted by oncologists.
Ihara et al., 2012 [25]	Japan	Comparative pre-post study	*n* = 33 breast cancer patients in 2008 and *n* = 27 breast cancer patients in 2007	Assessment of pharmacists’ interventions regarding prevention of chemotherapy-induced nausea and vomiting (CINV) in breast cancer patients receiving anthracycline and cyclophosphamide.	The efforts of pharmacists led to an improvement in therapeutic efficiency concerning the number of patients and hospital revenue. Additionally, appropriate use of antiemetics resulted in enhanced control of CINV and was cost-effective.
Jones et al., 2012 [32]	USA	Retrospective cohort study	*n* = 145 patients with breast cancer who received warfarin therapy for venous thromboembolism (VTE)	Assessment of clinical outcomes related to the quality of pharmacist-managed anticoagulation care with warfarin in patients with breast cancer.	Pharmacist-led anticoagulation service led to lower rates of recurrent VTE, and bleeding events compared to other oncology patients in the published literature.
Khadela et al., 2022 [22]	India	Prospective, single-centered pre-post study	*n* = 105 (54 controls and 51 cases, all diagnosed with breast cancer)	Assessment of the change in QALYs after providing oncology pharmacist’s services to assess its impact on the humanistic outcome.	Significant improvement in QALYs after the provision of oncology pharmacist services resulted in significant improvement.
Leenhardt et al., 2021 [27]	France	Prospective clinical trial	*n* = 51 breast cancer patients	Evaluation of the relevance and impact of pharmacist consultation and drug-drug interactions (DDI) management during the inclusion step of a clinical trial.	Pharmaceutical invention has reduced the risk of DDI in one-third of patients.
Li et al., 2021 [26]	USA	Randomized controlled trial	*n* = 613 cancer patients of whom 136 with breast cancer	Evaluation of efficacy of Specific Geriatric Assessment-Driven Intervention (GAIN) in reducing chemotherapy-related toxic effects in older adults with cancer.	Integration of multidisciplinary GAIN involving oncologist, nurse practitioner, social worker, physical/occupation therapist, nutritionist, and pharmacist, significantly reduced grade 3 or higher chemotherapy-related toxic effects in older adults with cancer.
Muluneh et al., 2018 [13]	USA	Interventional pre-post study	*n* = 107 (18 patients with breast cancer)	Effectiveness of a closed-loop, pharmacist-led oral chemotherapy management program	Clinical pharmacists’ services led to better patient knowledge regarding oral chemotherapy and improved adherence rates.
Neuner et al., 2022 [23]	USA	Single-arm, pre-post intervention study	*n* = 18 patients with stage I-III breast cancer.	Assessment of clinical pharmacist-led symptom monitoring and management intervention to improve adherence to endocrine therapy.	Forty-four percent of the patients became adherent after clinical pharmacist intervention. Additionally, improvement in patient-reported outcome assessments (physical, mental, social health, and self-efficacy) was reported.
Patel et al., 2023 [31]	USA	A quality improvement project	*n* = 53 patients with breast cancer	Evaluation of the impact of an outpatient pharmacy team-led intervention on treatment delays in medication initiation and adherence assessment.	Incorporation of pharmacists in outpatient oncology clinic visits was associated with decreased treatment day delays. Pharmacists conducted 31 adherence counseling interventions.
Rabeea et al., 2023 [34]	Iraq	Case-control study	*n* = 75 patients with breast cancer	Investigation of the effect of pharmacist intervention in optimizing adherence to oral hormone therapy.	Pharmacist involvement through patient education and follow-up had a significant impact on optimizing adherence to adjuvant hormone therapy.
Tanaka et al., 2018 [28]	Japan	Cross-sectional, questionnaire-based study	*n* = 39 patients with breast cancer	Investigation of the influence of adverse event change by pharmacist counseling on the QoL of outpatients receiving breast cancer chemotherapy.	Pharmacists can improve chemotherapy outpatients’ QoL regarding malaise and nausea by using personal counseling.
Tanaka et al., 2019 [29]	Japan	Cost-utility pharmacoeconomic analysis	*n* = 38 patients with breast cancer	Economic evaluation of pharmacist consultations with patients undergoing outpatient chemotherapy for breast cancer.	Pharmacists’ counseling had an acceptable ICER, contributing to improved patient QoL without significant additional expenditure on healthcare.

Abbreviations: CINV, chemotherapy-induced nausea and vomiting; DDI, drug-drug interactions; GAIN, Geriatric Assessment-Driven Intervention (GAIN); QoL, quality of life; VTE, venous thromboembolism.
